# Dietary protein restriction inhibits tumor growth in human xenograft models of prostate and breast cancer

**DOI:** 10.18632/oncotarget.1586

**Published:** 2013-11-23

**Authors:** Luigi Fontana, Remi M. Adelaiye, Antonella L. Rastelli, Kiersten Marie Miles, Eric Ciamporcero, Valter D. Longo, Holly Nguyen, Robert Vessella, Roberto Pili

**Affiliations:** ^1^ Department of Medicine, Washington University in St. Louis, MO, USA; ^2^ Department of Medicine, Salerno University Medical School, Salerno, Italy; ^3^ CEINGE Biotecnologie Avanzate, Napoli, Italy; ^4^ Genitourinary Program, Roswell Park Cancer Institute, Buffalo NY; ^5^ Department of Cancer Pathology and Prevention, Roswell Park Cancer Institute Division, University at Buffalo, NY; ^6^ Medicine and Experimental Oncology, University of Turin, Turin Italy; ^7^ Longevity Institute School of Gerontology and Department of Biological Sciences, University of Southern California, Los Angeles, CA, USA; ^8^ Department of Urology, University of Washington Medical Center, USA

**Keywords:** protein restriction, mTOR, prostate and breast cancer

## Abstract

Purpose: Data from epidemiological and experimental studies suggest that dietary protein intake may play a role in inhibiting prostate and breast cancer by modulating the IGF/AKT/mTOR pathway. In this study we investigated the effects of diets with different protein content or quality on prostate and breast cancer.

Experimental Design: To test our hypothesis we assessed the inhibitory effect of protein diet restriction on prostate and breast cancer growth, serum PSA and IGF-1 concentrations, mTOR activity and epigenetic markers, by using human xenograft cancer models.

Results: Our results showed a 70% inhibition of tumor growth in the castrate-resistant LuCaP23.1 prostate cancer model and a 56% inhibition in the WHIM16 breast cancer model fed with a 7% protein diet when compared to an isocaloric 21% protein diet. Inhibition of tumor growth correlated, in the LuCaP23.1 model, with decreased serum PSA and IGF-1 levels, down-regulation of mTORC1 activity, decreased cell proliferation as indicated by Ki67 staining, and reduction in epigenetic markers of prostate cancer progression, including the histone methyltransferase EZH2 and the associated histone mark H3K27me3. In addition, we observed that modifications of dietary protein quality, independently of protein quantity, decreased tumor growth. A diet containing 20% plant protein inhibited tumor weight by 37% as compared to a 20% animal dairy protein diet.

Conclusions: Our findings suggest that a reduction in dietary protein intake is highly effective in inhibiting tumor growth in human xenograft prostate and breast cancer models, possibly through the inhibition of the IGF/AKT/mTOR pathway and epigenetic modifications.

## INTRODUCTION

Prostate (PCa) and breast (BC) cancers are the most commonly diagnosed cancer in men and women living in Western countries [1]. Studies of populations migrating from low- to high-risk areas have shown a steep rise in PCa and BC rate [2,3]. In addition, in the last three decades the age-standardized PCa and BC incidence and mortality rate has increased dramatically in Japan and Singapore, two developed countries previously considered having a very low prevalence rate [4,5]. These studies strongly suggest that environmental factors play a key role in PCa and BC pathogenesis. It has been hypothesized that this increased prevalence of PCa and BC is partially due to the radical dietary shifts from traditional to Western diet patterns [2,6], which are characterized by high intakes of animal protein and fats, and refined carbohydrates.

Data from epidemiological and experimental studies indicate that protein intake is one of the most important dietary regulators of circulating levels of IGF-1, a powerful growth factor, which activates the Akt/mTOR pathway [7,8]. High circulating levels of IGF-1 are associated with increased risk of PCa and BC [9-11], Moreover, multiple lines of evidence have shown that activation of the PI3K/AKT/mTOR pathway, through insulin/IGF-1 stimulation and/or high levels of essential amino acids, play a crucial role in maintaining the malignant phenotype, and its inhibition antagonizes growth and motility of a range of cancer cells in mouse models [12-17].

In this study, we assessed whether a reduction of protein intake or modifications in aminoacid composition of isocaloric diets could inhibit PCa growth by using the LuCaP23.1 androgen-sensitive and castrate-resistant patient-derived xenograft model. LuCaP23.1 represents a relevant model for studying therapeutic interventions in a preclinical setting because it retains major clinical hallmarks of human PCa, including heterogeneous growth, prostate specific antigen (PSA) production, androgen-responsiveness, and resistance to castration [18]. In addition, we assessed whether or not protein intake could also inhibit BC growth by using the breast cancer cell line WHIM16. Finally, we investigated whether these dietary manipulations could modulate IGF-1 production, mTOR activity, cell proliferation, and key epigenetic markers of PCa progression, such as the methyltransferase EZH2 and associated histone mark H3K27me3 [19,20].

## RESULTS

### Protein restriction inhibits tumor growth in human prostate and breast cancer models

**Figure 1:**
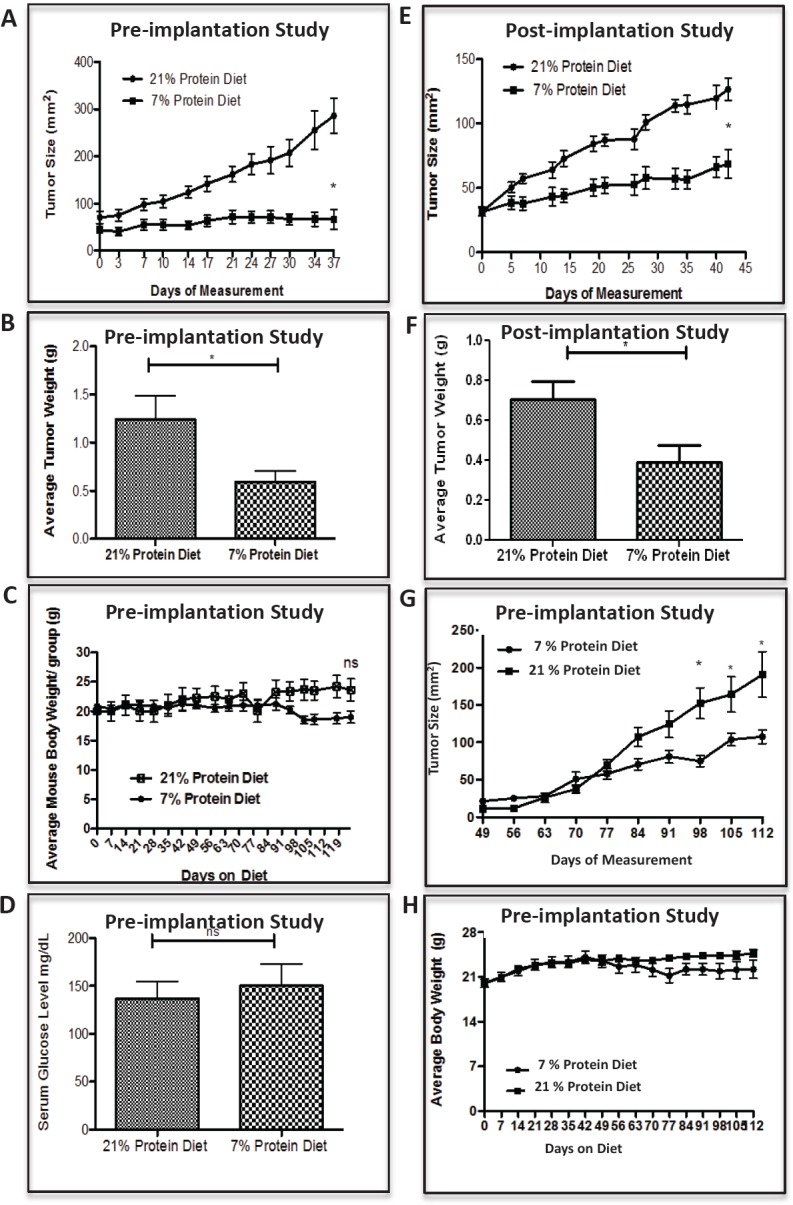
Low protein diet attenuates the growth of prostate and breast cancer in the castrate-resistant LuCaP23.1 model and in the WHIM16 model, respectively Tumor sizes were assessed two times a week by caliper measurements. (A, B) LuCaP23.1-CR and (G) WHIM16 growth curve of tumors already exposed to low protein diet (pre-implantation studies) and endpoint tumor weights. (E, F) LuCap23.1-CR growth curve of tumors exposed to low protein diet after tumors were implanted and established (post-implantation studies), and endpoint tumor weights. (C) LuCaP23.1-CR and (H) WHIM16 mouse body weights. (D) Measurements of serum glucose in LuCaP23.1-CR bearing mice. Results are expressed as the mean +/− SE, n= 7-10; * p<0.05.

To test the hypothesis whether a isocaloric decrease in dietary protein intake inhibits tumor growth in a human animal model of PCa and BC, we first designed and tested murine diets containing the lowest concentrations of protein that did not result in weight loss or health impairment. These studies showed that an “ad libitum” fed diet providing 7% calories from protein provided the lowest protein level compatible with health and weight maintenance (data not shown). In our first experiment (pre-implantation study), we acclimatized 4-6 week old male SCID mice to either the 21% or 7% protein diet for 4 weeks, prior to surgical castration and subcutaneous implantation of LuCaP23.1-CR tumors. As shown in figure 1A, LuCap23.1-CR xenograft growth was strikingly reduced in the 7% than in the 20% protein diet group, resulting in a 70% (p< 0.001, 95% CI= 55.98 to 139.7) reduced tumor size at 5 weeks post tumor implantation. Consistently, average tumor weight at the end of the experiment was 81% (p<0.0009, 95% CI =0.3814-1.243) lower in the 7% protein than in the 20% protein diet group (Fig. 1B). In a second experiment (post-implantation study), protein restriction was initiated in castrated mice 4 weeks after tumor establishment (~50 mm^2^). As shown in figure 1E, also in this setting the 7% protein diet markedly inhibited tumor growth and resulted in a ~50% (p<0.0275, 95% CI = 0.04232-0.5910) reduction in tumor weight (Fig.1F). Throughout the 4-month study, there was no significant difference in mean body weights between the 7% and the 21% protein diet groups (Fig. 1C). Interestingly, despite the higher carbohydrate content of the 7% protein diet, there was no significant difference in serum glucose concentration between the two groups (Fig. 1D).

To test the hypothesis whether an isocaloric decrease in dietary protein intake inhibits tumor growth in a human animal model of BC as well, we acclimatized 4-6 week old female NOD-SCID mice to either the 21% or 7% protein diet for 4 weeks, prior to subcutaneous implantation of WHIM16 tumor cells. As shown in figure 1G, WHIM16 xenograft growth was significantly reduced in the 7% as compared to the 20% protein diet group, resulting in a 56% (p< 0.0232, 95% CI= −153.9 to −12.86) reduced tumor size. No significant difference in mean body weights between the 7% and the 21% protein diet groups was observed (Fig. 1H).

### Protein restriction reduces serum IGF-1 concentration and attenuates mTOR activity in a human castrate-resistant, prostate cancer model

**Figure 2:**
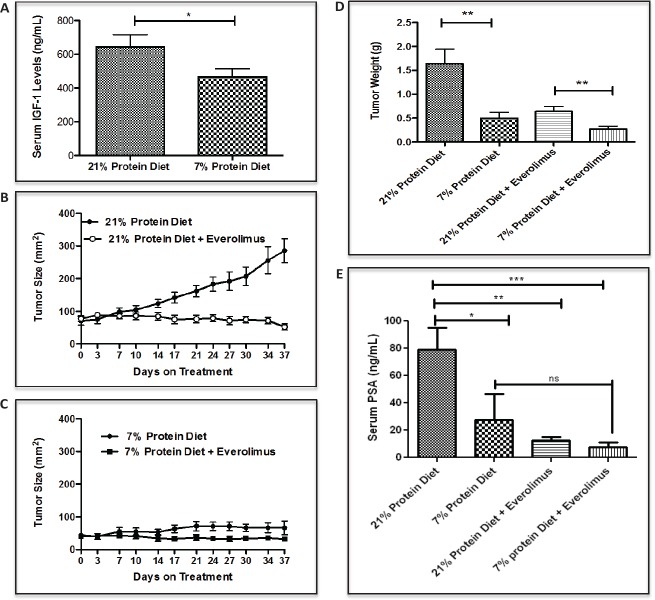
Low protein diet decreases IGF-1 serum levels and inhibit LuCaP23.1-CR growth in combination with everolimus (A) Measurements of serum IGF-1 in LuCaP23.1-CR bearing animals fed with either 21% or 7% protein diet. (B, C) Mice were acclimatized for four weeks to either 21% or 7% protein diet and after LuCaP23.1-CR xenograft implantation were treated with everolimus (2 mg/kg PO, daily X5 times/week). (D, E) Endpoint PSA and tumor weights were collected. Results are expressed as the mean +/− SE, n=10.

**Figure 3:**
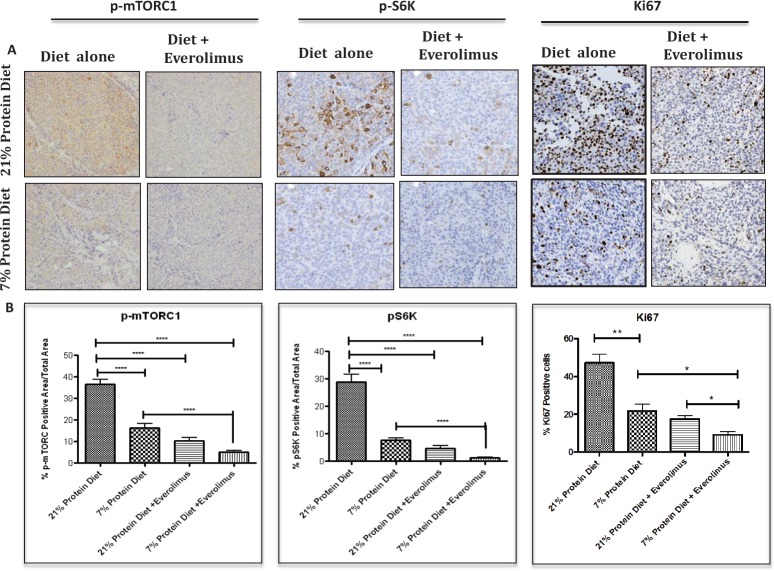
Low protein diet decreases mTOR and proliferation activity in the LuCaP23.1-CR model (A) At the end of the “pre-implantation” experiment, tumor samples were collected and processed. Paraffin embedded tissue specimens were stained for p-mTOR, p-S6 ribosomal protein and proliferation marker Ki67. (B) Quantification of staining. Results are based on four fields per tissue and are expressed as the mean +/− SE. **p<0.01 and ***p<0.001.

To determine whether dietary protein intake affects circulating IGF-1 levels in castrated tumor bearing mice, serum IGF-1 concentration were measured in the two groups. Serum IGF-1 concentration was significantly lower in the 7% than in the 21% protein diet group (Fig. 2A). As both serum IGF-1 and amino acids levels are important regulators of mTORC1 activity, we were interested in determining whether or not a reduction in protein intake down-regulates the mTOR signaling pathway in the LuCaP23.1-CR model, and whether or not the diet-induced inhibitory effect on tumor growth was dependent on mTOR inhibition. To test this hypothesis, we assessed the effects on tumor growth and the levels of phosphorylated mTOR and p70 S6K in mice treated with diet alone versus diet in combination with everolimus treatment. As shown in figure 2B and C, everolimus treatment was as effective as protein restriction in inhibiting tumor growth and had greater effect in combination with protein restriction as compared to lower protein diet alone. Tumor weights from the animals treated with everolimus alone or the 7% protein diet alone were similar (Fig. 2D). However, tumor weights were significantly smaller in animals treated with everolimus fed the 7% protein diet than in those treated with everolimus fed the 20% protein diet, suggesting that protein restriction and mTOR inhibition have additive inhibitory effects on PCa development. Endpoint PSA measurements confirmed the inhibitory effect of protein restriction alone and everolimus in both the 21% and 7% protein diet groups (Fig. 2E). Immunohistochemistry staining showed a significant decrease in phospho-mTORC1 levels following protein restriction (21% protein diet =37% +/− 2.09 vs. 7% protein diet=19% +/− 2.03, p<0.0001, 95% CI = 14.51- 26.19), which was even more pronounced when protein restriction was combined with everolimus (Fig. 3A and B). We also observed a similar significant decrease in ribosomal protein TOR activity as indicated by the down-regulation of p70 S6K (21% protein diet =29%+/− 2.74 vs. 7% protein diet = 8%+/− 0.84, p<0.0001, 95% CI= 15.4-27.34) in mice fed the 7% protein diet, which is potentiated by everolimus treatment. Interestingly, associated with the reduced mTOR activity we observed a decreased number of proliferating cells as indicated by the Ki67 staining (21% protein diet = 47% +/− 4.5 vs. 7% protein diet = 22% +/− 3.4, p<0.0041, 95% CI= 11.55- 39.10) (Fig. 3A and B). Protein diet restriction induced a similar inhibition of mTOR pathway and Ki67 expression in another patient-derived xenograft model of PCa (supplementary Fig. S1).

### Protein restriction inhibits tumor growth in a human androgen-sensitive prostate cancer model

To determine whether the androgen status could affect the inhibitory effects of protein restriction on PCa growth, we acclimatized the mice to either 21% or 7% protein diet and after four weeks we inoculated LuCaP23.1-AS tumors orthotopically in the prostate of non-castrated SCID mice. The 7% protein diet had a dramatic inhibitory effect on the growth of AS tumors implanted orthotopically in the prostate of intact animals (supplementary Fig. S2). Interestingly, the effects of protein restriction on average tumor weight was identical in the animal fed with a 7% diet alone and in those fed a 21% protein diet treated with everolimus.

### Protein restriction induces epigenetic modifications

**Figure 4:**
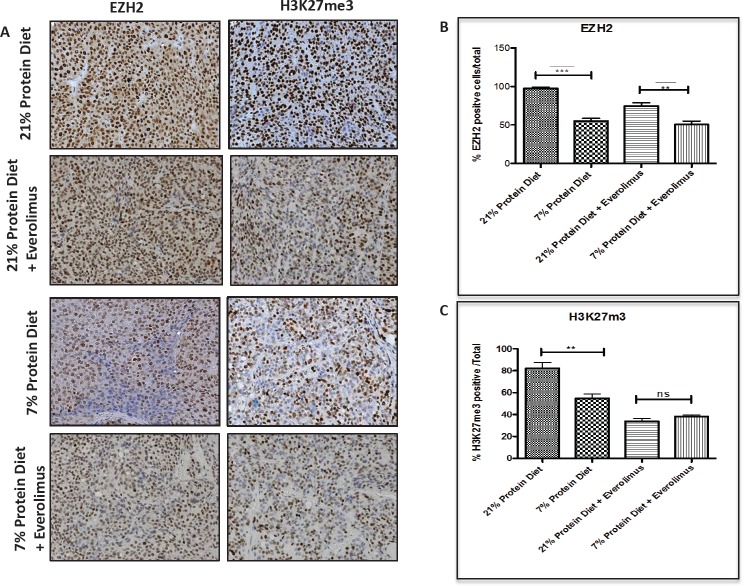
Epigenetic alterations associated with low protein diet in the LuCaP23.1-CR model (A) At the end of the “pre-implantation” experiment, tumor samples were collected and processed. Paraffin embedded tissue specimens were stained for the histone methyltransferase EZH2 and the associated histone mark H3K27me3. (B) Quantification of staining. Results are based on four randomly selected fields and are expressed as the mean + SE. **p<0.01, ***p<0.001

Because in our study the tumor inhibitory effects of protein restriction were partially independent of mTOR, we assessed whether epigenetic modifications, that are known to be associated with PCa progression, were modified by protein restriction in the LuCaP23.1-CR model. We found that the mice fed with a 7% protein diet had significantly lower expression of EZH2 (21% protein diet = 97.3% +/− 4.4 vs. 7% protein diet = 55.1% +/− 3.46, p<0.0001, 95% CI= 32.8- 51.65) and H3K27me3 (21% protein diet =82.3% +/− 4.4 vs. 7% protein diet= 54.9% +/− 3.8, p<0.0051, 95% CI= 11.83- 43.02) (Fig. 4A, B and C). Similar changes in EZH2 and H3K27me3 were also observed in mice treated with everolimus.

### Plant protein diets inhibit tumor growth independently of protein content

**Figure 5:**
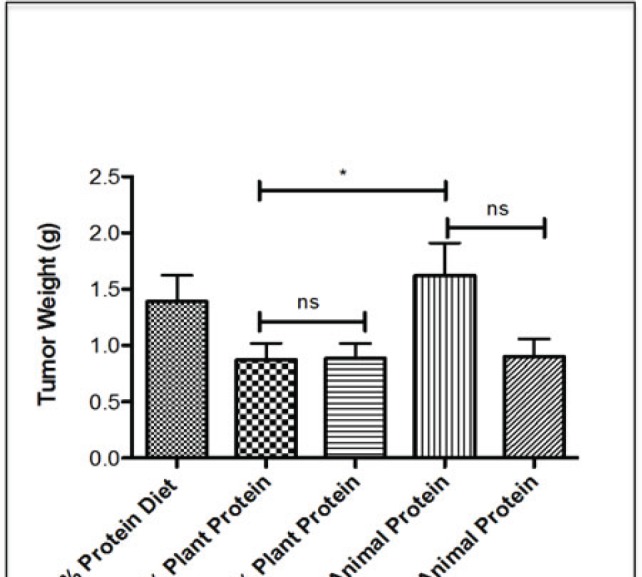
Effect of the different sources of protein diet on tumor growth and potential molecular mechanisms Endpoint LuCaP23.1-CR tumor weights of mice fed with either 21% protein diet (which consist of both plant and animal protein sources), 20% or 10% protein diet (plant based) or 20% or 10% protein diet (animal based). Results are presented as a mean +/− SE, n= 7-8 per group. *p<0.05, ns= not statistically significant.

To investigate whether modifications in protein quality (i.e. aminoacid composition) are beneficial in delaying PCa growth, mice were placed on either regular (20%) or a reduced (10%) protein diet from animal or vegetable sources for 4-weeks prior to surgically castration and subcutaneous implantation of LuCaP23.1-CR xenografts. At the end of the experiment (6 weeks), mice were sacrificed and tumor weights were collected. As shown in figure 5, a diet containing 20% plant protein decreased tumor weight by 37% as compared to a 20% animal dairy protein diet (p<0.045, 95% CI= −1.484 to −0.01818). The inhibitory effect on tumor growth exerted by the 20% and 10% plant protein diets was similar. Interestingly, there was no additive effect of switching from animal to plant proteins when dietary protein content was 10%, suggesting that a threshold exists below which the amino acid composition is less important than the protein content of the diet.

## DISCUSSION

In this study we examined the effects of isocaloric modifications in dietary protein quantity or quality on tumor growth in the human xenograft LuCaP23.1 (CR and AS), LuCaP35V (CR) and WHIM16 BC models. Our findings indicate that dietary protein reduction results in a marked inhibition of PCa and BC growth. This inhibitory effect was associated with a reduction in serum PSA and IGF-1 levels, and a down-regulation of intratumor mTOR activity in the LuCaP23.1 LuCaP35V models. Protein restriction was also associated with modulation of specific histone markers suggesting epigenetic modulation. Interestingly, the source of proteins appears to be important, as tumor inhibition was also achieved by changes in the quality (i.e. aminoacid composition of vegetable versus dairy) of protein food.

Accumulating scientific data indicate that perturbations in the IGF/PI3K/Akt/mTOR pathway play a key role in the pathogenesis of PCa and BC [10-18,24]. A linear relationship exists between circulating IGF-1 levels and the risk of developing PCa and BC [9,10,24], and the PI3K/Akt/mTOR signaling pathways is upregulated in 30-50% of PCa [12,13]. Data from epidemiological and human experimental studies suggest that dietary protein or essential amino acid restriction is more powerful than calorie or fat restriction in lowering the circulating levels of IGF-1, which in turn inhibits the PI3K/AKT/mTOR pathway [7,8,21,23,24]. Consistently, our data show that an isocaloric reduction of protein intake significantly reduces serum IGF-1 concentrations, inhibits mTOR activity, as indicated by a down-regulation of phosphorylated mTOR and p70-S6K, and reduces cell proliferation as indicated by the decrease in Ki67. In addition, our data show that a reduction of diet protein intake from 21% to 7% inhibits PCa and BC growth by 70% or more both when tumors where implanted 4 weeks before (pre-implantation studies) or 4 weeks after (post-implantation studies) the implementation of protein restriction. This inhibition in PCa growth induced by protein restriction appears to be greater than the effect induced by fat or carbohydrate restriction previously reported by other groups [25-27], and similar to the inhibitory effect induced by calorie restriction [28]. We also observed an additive effect of protein restriction and pharmacological mTOR inhibition (i.e. everolimus treatment) on mTOR activity, cell proliferation and PCa growth, suggesting that protein restriction works through multiple pathways and molecular targets. Interestingly, LuCaP23.1 is a PTEN expressing tumor, but with intrinsic mTOR pathway activation. Ongoing studies with PCa derived from genetically modified mice will determine the importance of mTOR activity in the sensitivity to protein restriction.

At least 15-17% (i.e. ~1.3-1.5 g/kg/body weight) of the total calories consumed by US and Northern European citizens comes from dietary protein, which is two-fold higher than what is recommended by the USDA (i.e. recommended daily allowance, 0.85 g/kg/body weight or 10% protein from calories) and of the quantity consumed (9% calorie from protein) by the Okinawan centenarians who had one of the lowest rate of PCa and BC in the world [29-32]. Moreover, the great majority of proteins in the typical Western diet come from meat, egg and dairy food sources, which are very rich in essential amino acids. Elevated levels of essential amino acids are able to fully activate, independently of IGF-1-insulin signaling, the TORC1 complex, which regulates cell growth, protein synthesis, and autophagy [12]. Our data suggest that feeding mice isocaloric and isoproteic diets with lower concentrations of some essential aminoacids (i.e. isoleucine, lysine, tyrosine, threonine, tryptophan, valine and methionine) reduces PCa growth, though at a lesser extent than protein restriction. Our experimental findings support the notion that the high intake of animal (dairy) proteins in Western diets may play a role in PCa development and progression, whereas more traditional diets rich in proteins from cereals and legumes might partially inhibit PCa growth [33].

**Figure 6:**
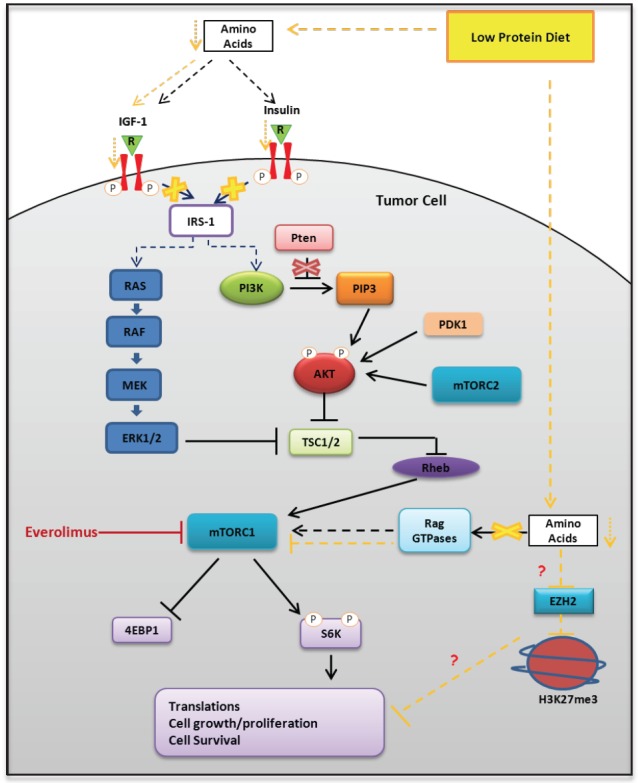
Representative schema of the potential molecular mechanisms responsible for the biological effects of protein restriction

Epigenetics is a potential mechanistic link between diet, energy metabolism, and gene expression modulation [34,35]. Highly accessible chromatin or open chromatin is typically observed at active regulatory regions like enhancers and promoters. Closed chromatin, on the other hand, is observed at silenced regions like hyper-methylated promoters of repressed genes. Diet and energy metabolism affect epigenetic enzymes that regulate histone marks [34-36]. Enhancer of zeste homolog 2 (EZH2), a subunit of Polycomb repressive complex 2, inhibits gene expression via its histone methyltransferase activity. A recent report has shown that the oncogenic function of EZH2 in cells of CR prostate cancer is independent of its role as a transcriptional repressor and involves its the ability to act as a coactivator for critical transcription factors including the androgen receptor [16]. Interestingly, in our study we observed that protein restriction decreased the expression of EZH2 and its associated histone repressive marker H3K37me3. This observation suggests that protein restriction is responsible for selective epigenetic changes that may drive modulation specific pathways involved in PCa growth and survival (Fig. 6). Additional studies are needed to shed light on the specificity of the histone markers and associated gene expression modulation following protein restriction.

The results from our studies may have a rapid translation into the clinic. We envision that patients with recurrent PCa and BC could be offered diet modifications involving protein dietary content at different stages of their disease with appropriate monitoring. The absence of a differential effect in the AS and CR LuCaP23.1 models suggest that the androgen status does not affect the response of PCa to protein restriction. Furthermore, intratumor androgen receptor expression was not inhibited in the 7% protein diet group (data not shown). An ongoing clinical study in patients with newly diagnosed PCa will provide important information on the biological changes following this dietary intervention before prostatectomy.

In summary, a high-protein diet promoted the growth of human LuCaP23.1 and WHIM16 tumors in mice, and a reduction in protein intake resulted in a significant inhibition of tumor growth even when the diet was started after the formation of measurable tumors, possibly through inhibition of the IGF/Akt/mTOR pathway and epigenetic modifications. Moreover, our findings indicate that plant proteins partially inhibit prostate cancer growth independently of caloric or proteic intake, suggesting that protein quality plays a key role in the progression of prostate and breast cancer. Clinical studies in patients with prostate and breast cancer are warranted to confirm the impact of dietary modifications in protein content and quality on tumor progression.

## MATERIALS AND METHODS

### Androgen-sensitive and castrate-resistant LuCaP23.1 xenograft models of human prostate cancer

Six-weeks old, male homozygous Icr SCID mice were purchased from the in-house animal resource core facility Roswell Park Cancer Institute (Buffalo, NY). Mice were housed and maintained in a sterile and pathogen free facility, in accordance with the Institutional guidelines credited by the Institutional Animal Care and Use Committee. Castrate-resistant (CR) LuCaP23.1 xenografts were generated by serial passages of androgen-sensitive (AS) LuCaP23.1 tumors in surgically-castrated mice [18]. LuCaP23.1 AS and CR tumors were dissected into ~1 mm^2^ pieces and implanted either subcutaneously under the skin or orthotopically into the dorsal lobe of the prostate in both intact (LuCaP23.1-AS) or surgically-castrated (LuCaP23.1-CR) mice. Similar approach was used for LuCaP35V-CR. All mice were operated under sedation with oxygen, isoflurane and buprenorphine. Mice were randomly grouped and placed on either 21% or 7% protein diets prior to tumor implantation (n=16-20 per group) or after tumor implantation (n=7 per group) or placed on diets from different sources of protein (n= 7-8 per group).

### WHIM16 xenograft model of human breast cancer

Six-weeks old, female NOD.Cg-Prkdcscid Il2rgtm1Wjl/SzJ mice were purchased from the Jackson Labs. Mice were housed and maintained in a sterile and pathogen free facility. WHIM16 tumor cell line was generated from a patient with ER positive/PR negative/HER2 negative breast cancer (Washington University). This tumor cell line carries also a PI3K mutation with activation of the AKT/mTOR pathway (manuscript submitted). Five million WHIM16 cells were implanted subcutaneously under the skin. All mice were operated under sedation with oxygen, isoflurane and buprenorphine. Mice were randomly grouped and placed on either 21% or 7% protein diets prior to tumor implantation (n=10 per group).

### Feeding protocol and drug treatment

The six experimental diets were prepared and sterilized by irradiation by Harlan Laboratories (Madison, WI). A summary of the composition and ingredients of each diet are shown in table 1. Animals were allowed free access to food in cage and autoclaved water supply via auto-watering system. Male mice that were randomized into 21% protein diet and 7% protein diet were further randomized within each group to either receive treatment with everolimus (10 mg/kg, 5 days on/2 days off) or vehicle.

**Table 1:** Composition and ingredients of experimental diets

	21% mix protein	7% mix protein		20% plant protein	10% plant protein		20% dairy protein	10% dairy protein
Diet composition			Diet composition			Diet composition		
Total energy value (kcal/g)	3.6	3.6	Total energy value (kcal/g)	3.7	3.7	Total energy value (kcal/g)	3.7	3.7
Carbohydrate (%Kcal)	58.9	73.0	Carbohydrate (%Kcal)	62.4	73.5	Carbohydrate (%Kcal)	63.6	74.0
Fat (%kcal)	20.1	20.2	Fat (%kcal)	17.6	16.6	Fat (%kcal)	16.4	16.0
Protein (%Kcal)	20.9	6.8	Protein (%Kcal)	20.0	10.0	Protein (%Kcal)	20.0	10.0
Leucine (g/kg)	25.4	8.8	Leucine (g/kg)	21.2	10.6	Leucine (g/kg)	18.7	9.4
Isoleucine (g/kg)	7.8	2.7	Isoleucine (g/kg)	7.9	4.0	Isoleucine (g/kg)	10.1	5.0
Lysine (g/kg)	16.3	4.0	Lysine (g/kg)	3.9	1.9	Lysine (g/kg)	15.7	7.9
Methionine (g/kg)	6.7	1.9	Methionine (g/kg)	4.1	2.1	Methionine (g/kg)	4.9	2.4
Cysteine (g/kg)	7.2	3	Cysteine (g/kg)	3.0	1.5	Cysteine (g/kg)	2.0	1.0
Arginine (g/kg)	6.3	2.9	Arginine (g/kg)	7.8	3.9	Arginine (g/kg)	6.7	3.3
Phenylalanine (g/kg)	6.6	2.4	Phenylalanine (g/kg)	10.4	5.2	Phenylalanine (g/kg)	8.8	4.4
Tyrosine (g/kg)	6.9	2.4	Tyrosine (g/kg)	5.0	2.5	Tyrosine (g/kg)	9.2	4.6
Histidine (g/kg)	3.4	1.4	Histidine (g/kg)	4.2	2.1	Histidine (g/kg)	4.9	2.4
Threonine (g/kg)	9.7	3.3	Threonine (g/kg)	5.5	2.7	Threonine (g/kg)	8.4	4.2
Tryptophan (g/kg)	3.4	1.0	Tryptophan (g/kg)	1.6	0.8	Tryptophan (g/kg)	2.5	1.2
Valine (g/kg)	8.4	3.2	Valine (g/kg)	8.7	4.3	Valine (g/kg)	11.8	5.9
								
Formula (g/kg)			Formula (g/kg)			Formula (g/kg)		
Corn	430	430	Wheat gluten	110	55	Casein	170	85
Lactalbumin	177	35	Corn gluten (60%)	136	68	Lactalbumin	44	22
DL-Methionine	2.0	0.4	Isolated soy protein	22	11			
Corn starch	149	287.4	Corn starch	325.4	459	Corn starch	380.1	486.3
Maltodextrin	100	100	Maltodextrin Sucrose	100 150	100 150	Maltodextrin Sucrose	100 150	100 150
Corn oil	29	32	Corn oil	32	32	Corn oil	32	32
Olive oil	29	32	Olive oil	32	32	Olive oil	32	32
Cellulose	30	30	Cellulose	50	50	Cellulose	50	50
* Mineral Mix, AIN-93G-MX	35	35	** Mineral Mix, w/o Ca & P	13.4	13.4	**Mineral Mix, w/o Ca & P	13.4	13.4
Calcium phosphate	8	8	Calcium phosphate Calcium carbonate	12 7.0	12.6 6.8	Calcium phosphate Calcium carbonate	8 10.3	10.8 8.3
***Vitamin Mix, Tekland	10	10	***Vitamin Mix, Teklad	10	10	***Vitamin Mix, Teklad	10	10

* Mineral Mix, AIN-93G-MX (No. 94046),

** Mineral Mix, w/o Ca & P (No. 98057),

*** Vitamin Mix, Teklad (40060),

### Tumor assessment

For tumors implanted subcutaneously, tumor sizes and body weights were recorded twice and once a week, respectively. Tumor weights were measured by using a weighing scale at the end of the experiments. Tumor sizes were assessed by caliper measurements of two diameters of the tumor (longest length × shortest length = mm^2^).

### Blood and prostate tissue collection, and IGF-1 measurement

At the end of the experiment, blood was drawn from all mice by cardiac bleed. Serum was separated, and aliquots were either used to assess levels of PSA and glucose in the serum or stored in −80°C for further analysis. Serum insulin growth factor 1 (IGF-1) concentration was measured in duplicates (n=8 per diet group) using a mouse specific IGF-1 ELISA kit (Abcam, Cambridge, MA, USA). Tumor tissues were excised, weighed and either stored in −80°C or fixed in 10% buffered formalin for histopathology.

### Immunohistochemistry

Tissue specimens were fixed for 24-hr, paraffin embedded and sectioned (4μm). Sections were de-paraffinized and rehydrated through graded alcohol washes. Antigen unmasking was achieved by boiling slides in sodium citrate buffer (pH=6.0). Sections were further incubated in hydrogen peroxide to reduce endogenous activity. To examine the expressions of our proteins of interests, tissue section were blocked with 2.5% horse serum (Vector Laboratories) and incubated overnight in primary antibodies against p-mTOR (1:400, Cell Signaling), p-S6K (1:200, Cell Signaling), Ki67 (1:50, Thermo Fisher), EZH2 (1:50, Cell Signaling), and H3k27me3 (1:200 Cell Signaling). Following primary incubation, tissue sections were incubated in horseradish-conjugated anti-rabbit or anti-mouse antibody according to manufacturer's protocol (Vector Laboratories) followed by enzymatic development in diaminobenzidine (DAB) and counter stained in hemotoxyline. Section were dehydrated and mounted with cytoseal 60 (Thermo Scientific). Stained sections were analyzed under bright field using the Zeiss Axio microscope. The number of positive cells was determined in a blinded fashion by analyzing four random 20× fields per tissue and quantified using Image J software.

### Statistical analysis

Quantitative measures were compared between groups (normal diet versus low protein or modified diets, with or without treatment) using two-tailed Student's *t*-test calculated by Graph-Pad software. The P values < 0.05 were considered to be statistically significant. The data are presented as mean ± SE.

## Supplementary Figures


