# Supplementary material for: Dietary protein restriction inhibits tumor growth in human xenograft models of prostate and breast cancer
Source: Oncotarget. 2013 Nov 23;4(12):2451–61. doi: 10.18632/oncotarget.1586 (PMC3926840; doi:10.18632/oncotarget.1586)
Supplement: Supplementary file 1 [file oncotarget-04-2451-s001.pdf]

Dietary protein restriction inhibits tumor growth in human xenograft models of prostate and breast cancer -Fontana et al

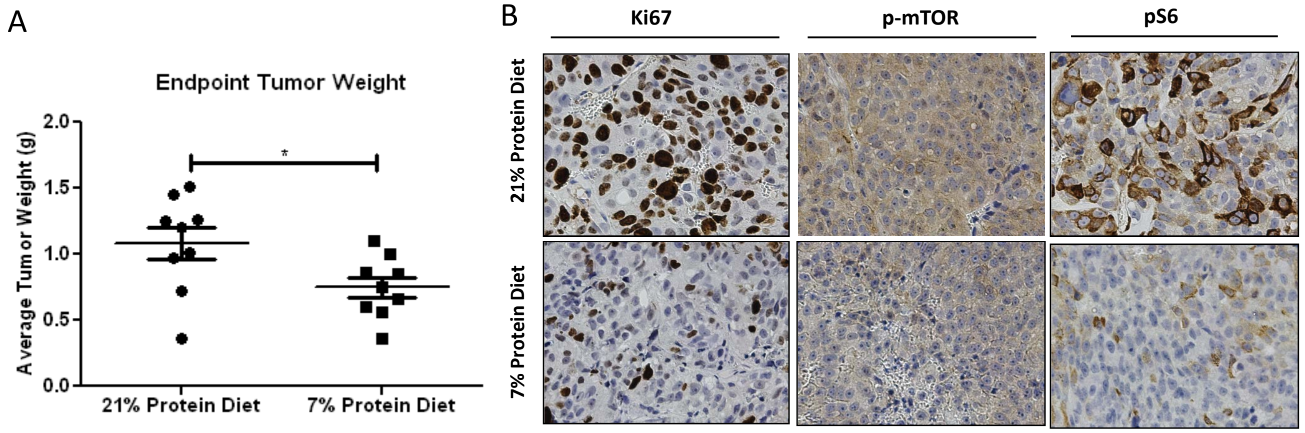

Supplementary Figure S1. Low protein diet attenuates the growth of PCa in the castration resistant LuCaP35V model. (A) Castrated male SCID mice were implanted with LuCaP 35V-CR tumor pieces subcutaneously. When tumors reached ~35 mm2, mice were placed on either 21% or 7% protein diet. Tumor weights were assessed. Results are expressed as the mean + SE, n=7-8. *p<0.005. (B) Paraffin embedded tissue specimens were stained for p-mTOR, p-S6 ribosomal protein and proliferation marker Ki67.

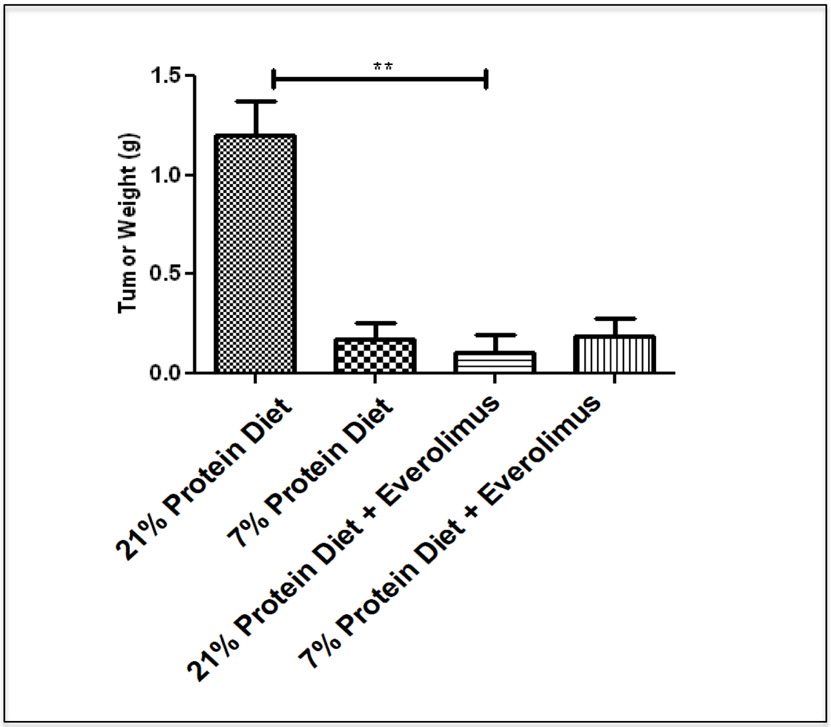

Supplementary Figure S2. Low protein diet attenuates the orthotopic growth of PCa in the androgen-sensitive LuCaP23.1 model. Mice were acclimatized for four weeks to either 21% or 7% protein diet and after LuCaP23.1-AS xenograft implantation were treated with everolimus (2 mg/kg PO, daily X5 times/week). Endpoint tumor weights were collected. Results are expressed as the mean + SE, n=10.
